# Correction: Aging-related upregulation of the homeobox gene *caudal* represses intestinal stem cell differentiation in *Drosophila*

**DOI:** 10.1371/journal.pgen.1011846

**Published:** 2025-09-04

**Authors:** Kun Wu, Yiming Tang, Qiaoqiao Zhang, Zhangpeng Zhuo, Xiao Sheng, Jingping Huang, Jie’er Ye, Xiaorong Li, Zhiming Liu, Haiyang Chen

S3 Fig was uploaded incorrectly. Please see the correct S3 Fig here.

## Supporting information

S3 Fig *Cad* functions in EBs to prevent ISCs to produce differentiated ECs. (A-B) Immunofluorescence images of *esg*-GFP (green) and Pdm1(red) staining with the midgut section from the R4 region of control *Drosophila* (A, *esg*^*ts*^-*Gal4* > *UAS-GFP*) and *Drosophila* carrying *esg*^*ts*^-*Gal4* > *UAS-cad* (B), under normal conditions. *esg*-GFP (green) represents ISCs and their differentiating cells. Pdm1 staining (red) was used to visualize matured ECs. (C) Quantification of the ratio of *esg*-GFP^+^ and Pdm1^+^ cells per 10,000 μm^2^ area of the midgut as indicated in (A-B). The number n represents counted ROIs in midguts from each experiment. Each dot corresponds to one ROI (10,000 μm^2^ area). (D-E) Immunofluorescence images of *NRE*-GFP (green) and Pdm1(red) staining with the midgut section from the R4 region of control *Drosophila* (D, *NRE*^*ts*^-*Gal4* > *UAS-GFP*) and *Drosophila* carrying *NRE*^*ts*^-*Gal4* > *UAS-cad* (B), under normal conditions. *NRE*-GFP (green) represents EBs. Pdm1 staining (red) was used to visualize matured ECs. (F) Quantification of the ratio of *NRE*-GFP^+^ and Pdm1^+^ cells per 10,000 μm^2^ area of the midgut as indicated in (D-E). The number n represents counted ROIs in midguts from each experiment. Each dot corresponds to one ROI (10,000 μm^2^ area). (G-H) Immunofluorescence images of *ISC*-GFP (*ISC*^*ts*^*-Gal4*-driven *UAS-GFP*; green) and Pdm1 (red) staining with the midgut section from the R4 region of control flies (G, *ISC*^*ts*^-*Gal4*-driven *UAS-GFP*) and *cad*-depleted *Drosophila* by *ISC*^*ts*^-*Gal4*-driven *cad* RNAi (H). *ISC*-GFP (green) indicates ISCs. Pdm1 staining (red) was used to visualize differentiating ECs. *ISC*-GFP^+^ and Pdm1^-^ cells are ISCs. *ISC*-GFP^-^ and Pdm1^+^ cells are mature ECs. (I) Quantification of the ratio of *ISC*-GFP^+^ and Pdm1^+^ cells per 10,000 μm^2^ area of the R4 region of midguts as shown in (G-H). The number n represents counted regions of interest in midguts from each experiment. Each dot corresponds to one region of interest (ROI = 10,000 μm^2^ area). (J-K) Immunofluorescence images of *ISC*-GFP (green) and Pdm1 (red) staining with the midgut treated with PQ-REC-1D. The midgut section from the R4 region of control flies (J, *ISC*^*ts*^-*Gal4*-driven *UAS-GFP*) and *cad*-overexpressing *Drosophila* by *ISC*^*ts*^-*Gal4*-driven *UAS-cad-HA* (K). *ISC*-GFP (green) represents ISCs. Pdm1 staining (red) was used to visualize differentiating ECs. White arrows indicate differentiating pre-ECs (*ISC*-GFP^+^ and Pdm1^+^cells). *ISC*-GFP^+^ and Pdm1^-^ cells are ISCs. *ISC*-GFP^-^ and Pdm1^+^ cells are mature ECs. (L) Quantification of the ratio of *ISC*-GFP^+^ and Pdm1^+^ cells per 10,000 μm^2^ area of R4 region midguts from control *Drosophila* and *cad*-overexpressing *Drosophila* carrying *ISC*^*ts*^-*Gal4*-driven *UAS-cad-HA* as shown in (J-K). The number n represents counted ROIs in midguts from each experiment. Each dot corresponds to one ROI (10,000 μm^2^ area). (M-O) Immunofluorescence analyses of control (*FRT40A*, M), *cad*^2^ mutant (N), and *cad-HA* overexpressing (O) mosaic analysis with repressible cell marker (MARCM) clones (green) 7 days after clone induction (ACI) of flies. Prospero staining (red) was used to visualize EEs. White arrows indicate EEs in clones. DAPI stained nuclei are shown in blue. Scale bars represent 10 μm (S3A, S3B, S3D, S3E, S3G, S3H and S3J–S3K Fig^‌‌^), 5μm (S3M–S3O Fig^‌‌^). Error bars represent SD. Student’s t-tests were used to assess statistical significance: *p < 0.05, **p < 0.01, ***p < 0.001, ****p < 0.0001, and NS (non-significant), which represents p > 0.05.
(TIF)
