# Supplementary figures and images for: Aging-related upregulation of the homeobox gene caudal represses intestinal stem cell differentiation in Drosophila
Source: PLoS Genet. 2021 Jul 6;17(7):e1009649. doi: 10.1371/journal.pgen.1009649 (PMC8284806; doi:10.1371/journal.pgen.1009649)

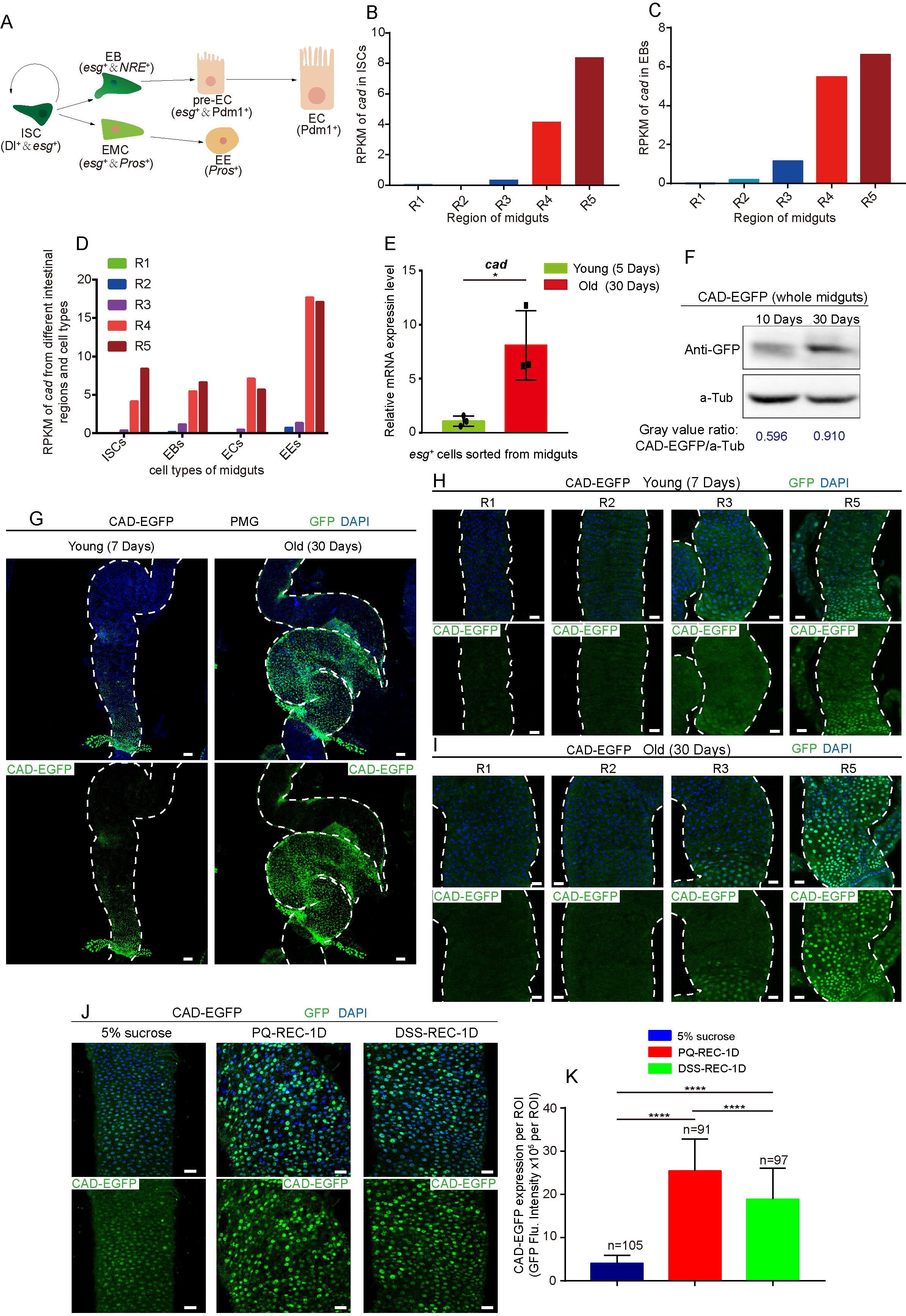

Supplement: S1 Fig — (A) Cartoon model of Drosophila ISC lineages. An ISC (Dl+ and esg+) undergoes asymmetric division once to produce a new ISC and a diploid precursor enteroblast (EB; esg+ and NRE+) or a diploid precursor enteroendocrine mother cell (EMC; esg+ and Pros+). The post-mitotic EB further differentiates into premature enterocytes (pre-ECs) (esg+ and Pdm1+), which continue to differentiate into octoploid mature ECs (Pdm1+). The EMC divides once to produce a pair of diploid enteroendocrine cells (EEs; Pros+). (B-C) The cad gene reads per kilobase per million mapped reads (RPKM) values in ISCs (B) and EBs (C) of five regions from the midguts of Drosophila. Expression was determined using RNAseq in experiments described in [44]. (D) The cad gene RPKM values in ISC, EB, EC, and EE from R1-R5 of midguts. Expression was determined using RNAseq in experiments described in [44]. (E) Relative mRNA fold change of cad in sorted esg-GFP+ cells of young (green bar) and aged (red bar) Drosophila (esg-GFP/CyO). The cad expressions in esg-GFP+ cells of Drosophila with different ages are plotted relative to 5-day Drosophila, which was set to 1. Error bars indicate the standard deviation (SD) of three independent experiments. (F) Western blot result of CAD expression in midguts from young (age 5 days) and old (age 30 days). (G) The whole posterior midgut immunofluorescence images of CAD-EGFP (green) staining from young (age 7 days) and old (age 30 days) Drosophila carrying CAD-EGFP (green). (H-I) Immunofluorescence images of CAD-EGFP (green) staining with the midgut section from R1-R5 region (except R4 which were showed in Fig 1) of young (7 days; H) and old (30 days; I) Drosophila carrying CAD-EGFP midgut. (J) Immunofluorescence images of CAD-EGFP (green) staining with the midgut section from the PMG (the posterior midgut) of young cad-EGFP Drosophila which were treated with 5% sucrose (used as control), PQ, or DSS, respectively. (K) Quantifications of fluorescence intensity of CAD-EGFP in p [file pgen.1009649.s001.tif]

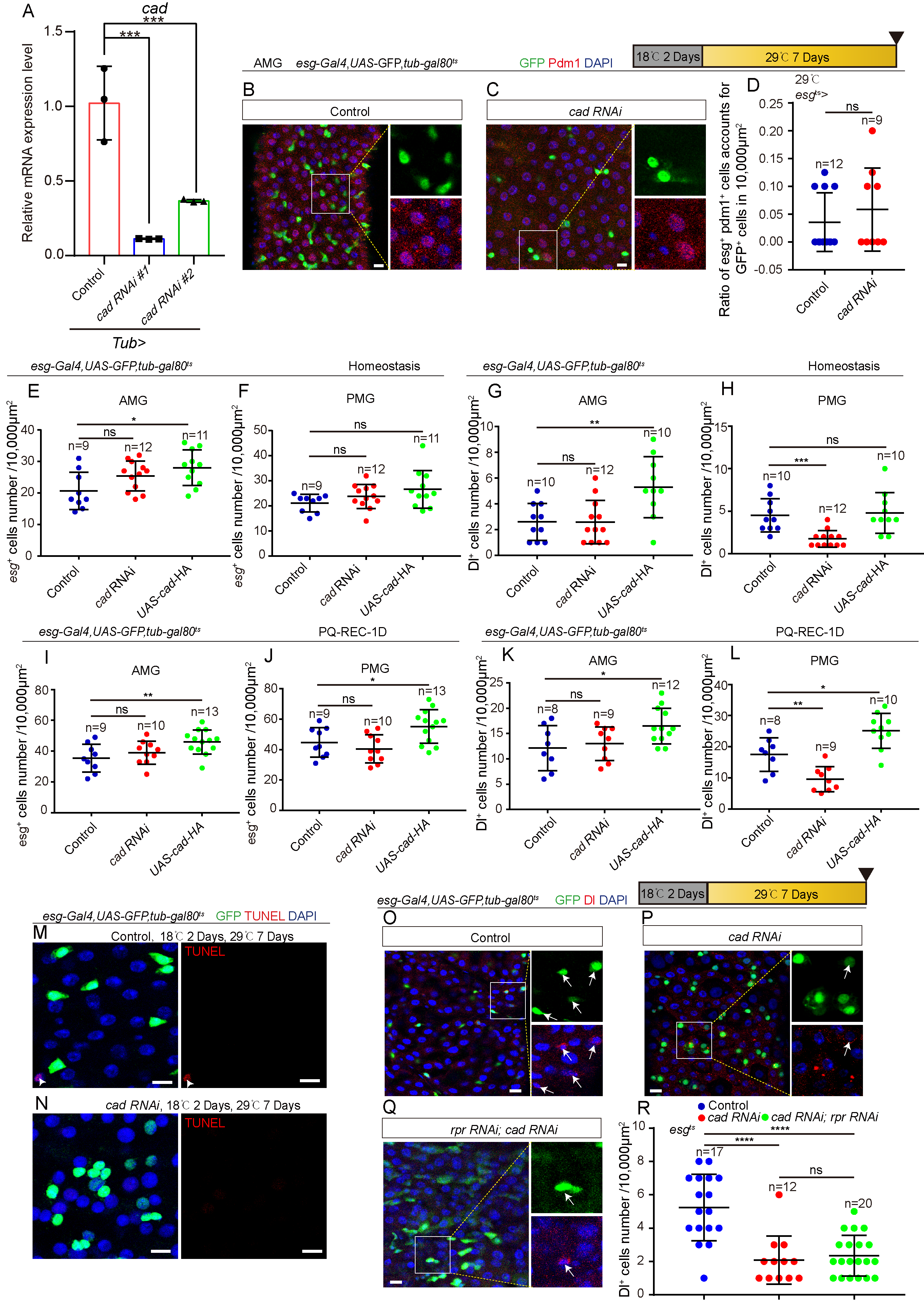

Supplement: S2 Fig — (A) Relative mRNA fold change of cad-depleted Drosophila by tub-Gal4-driven two different cad RNAi lines. The cad expressions in Drosophila with different RNAi are plotted relative to control flies (tub-Gal4), which was set to 1. Error bars indicate the standard deviation (SD) of three independent experiments. (B-C) Immunofluorescence images of esg-GFP (green) and Pdm1 (red) staining with the midgut under normal conditions. The midgut section from AMG of control flies (B, esgts-Gal4-driven UAS-GFP) and AMG of Drosophila carrying esgts-Gal4-driven cad RNAi (C). esg-GFP+ and Pdm1- cells are ISCs or EBs. esg-GFP- and Pdm1+ cells are matured ECs. (D) Quantification of the ratio of esg-GFP+ and Pdm1+ cells per 10,000 μm2 area of the AMG as indicated in (B-C). The number n represents counted ROI in midguts from each experiment. Each dot corresponds to one ROI (10,000 μm2 area). (E-H) Quantification of the esg+ cells (E-F) and the Dl+ cells (G-H) per 10,000 μm2 area of the anterior midgut (AMG; E and G) and the posterior midgut (PMG; F and H) from control Drosophila (esgts-Gal4-driven UAS-GFP), Drosophila carrying esgts-Gal4>cad RNAi, and Drosophila carrying esgts-Gal4>UAS-cad-HA, under homeostatic conditions. The number n is indicated. Each dot corresponds to one ROI (10,000 μm2 area). (I-L) Quantification of the esg+ cells (I-J) and the Dl+ cells (K-L) per 10,000 μm2 area of the AMGs (I and K) and the PMGs (J and L) from control Drosophila (esgts-Gal4-driven UAS-GFP), Drosophila carrying esgts-Gal4>cad RNAi, and Drosophila carrying esgts-Gal4>UAS-cad-HA, under PQ-treated conditions (PQ-REC-1D). The number n is indicated. Each dot corresponds to one ROI (10,000 μm2 area). (M-N) Immunofluorescence images of esg-GFP (green) and TUNEL (red) staining with the midgut under normal conditions. The midgut section from PMG of control flies (M, esgts-Gal4-driven UAS-GFP) and Drosophila carrying esgts-Gal4-driven cad RNAi (N). esg-GFP (green) identifies ISCs and their differentiatin [file pgen.1009649.s002.tif]

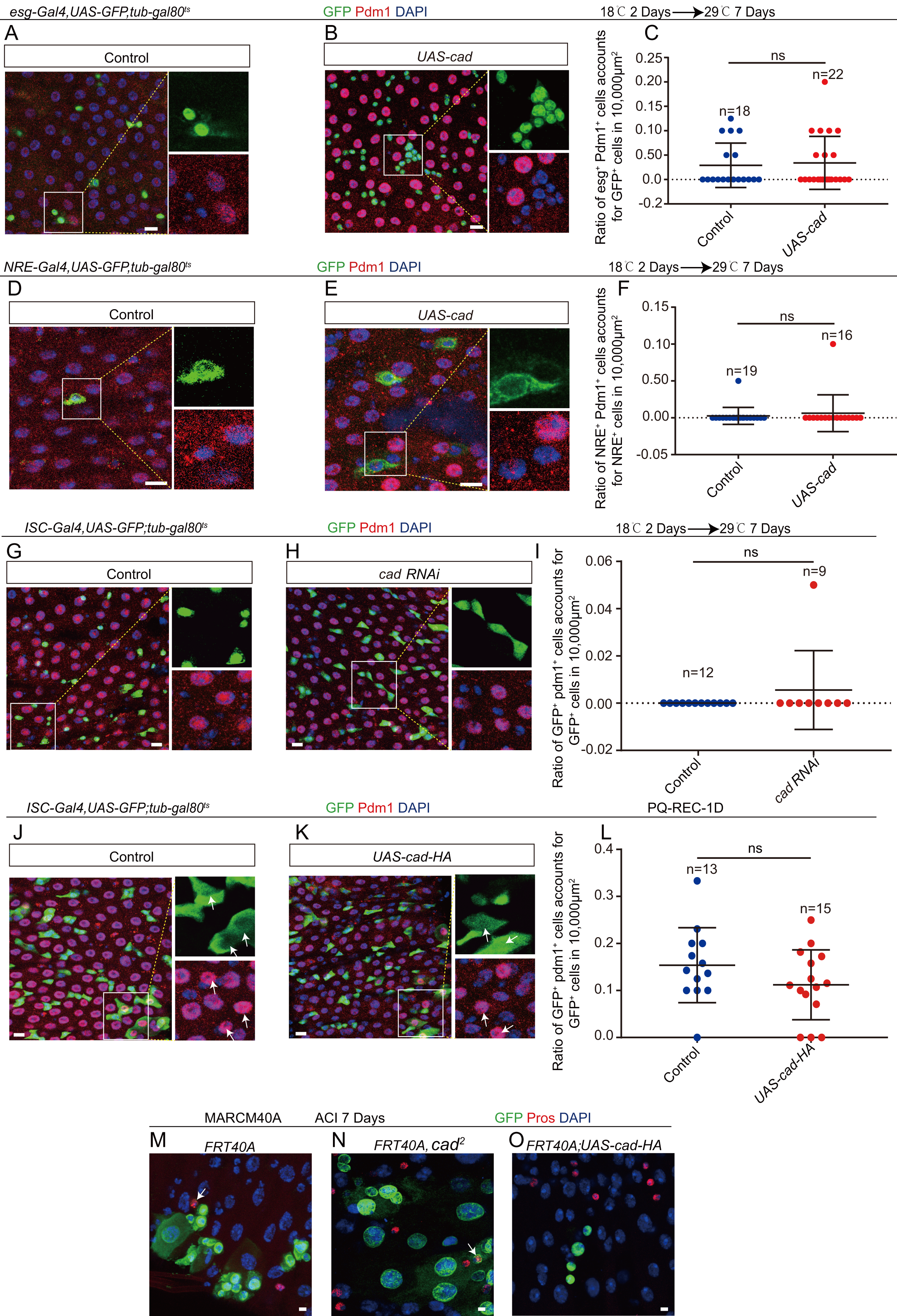

Supplement: S3 Fig — (A-B) Immunofluorescence images of esg-GFP (green) and Pdm1(red) staining with the midgut section from the R4 region of control Drosophila (A, esgts-Gal4>UAS-GFP) and Drosophila carrying esgts-Gal4>UAS-cad (B), under normal conditions. esg-GFP (green) represents ISCs and their differentiating cells. Pdm1 staining (red) was used to visualize matured ECs. (C) Quantification of the ratio of esg-GFP+ and Pdm1+ cells per 10,000 μm2 area of the midgut as indicated in (A-B). The number n represents counted ROIs in midguts from each experiment. Each dot corresponds to one ROI (10,000 μm2 area). (D-E) Immunofluorescence images of NRE-GFP (green) and Pdm1(red) staining with the midgut section from the R4 region of control Drosophila (D, NREts-Gal4>UAS-GFP) and Drosophila carrying NREts-Gal4>UAS-cad (B), under normal conditions. NRE-GFP (green) represents EBs. Pdm1 staining (red) was used to visualize matured ECs. (F) Quantification of the ratio of NRE-GFP+ and Pdm1+ cells per 10,000 μm2 area of the midgut as indicated in (D-E). The number n represents counted ROIs in midguts from each experiment. Each dot corresponds to one ROI (10,000 μm2 area). (G-H) Immunofluorescence images of ISC-GFP (ISCts-Gal4-driven UAS-GFP; green) and Pdm1 (red) staining with the midgut section from the R4 region of control flies (G, ISCts-Gal4-driven UAS-GFP) and cad-depleted Drosophila by ISCts-Gal4-driven cad RNAi (H). ISC-GFP (green) indicates ISCs. Pdm1 staining (red) was used to visualize differentiating ECs. ISC-GFP+ and Pdm1- cells are ISCs. ISC-GFP- and Pdm1+ cells are mature ECs. (I) Quantification of the ratio of ISC-GFP+ and Pdm1+ cells per 10,000 μm2 area of the R4 region of midguts as shown in (G-H). The number n represents counted regions of interest in midguts from each experiment. Each dot corresponds to one region of interest (ROI = 10,000 μm2 area). (J-K) Immunofluorescence images of ISC-GFP (green) and Pdm1 (red) staining with the midgut treated with PQ-REC-1D. The midgut section [file pgen.1009649.s003.tif]

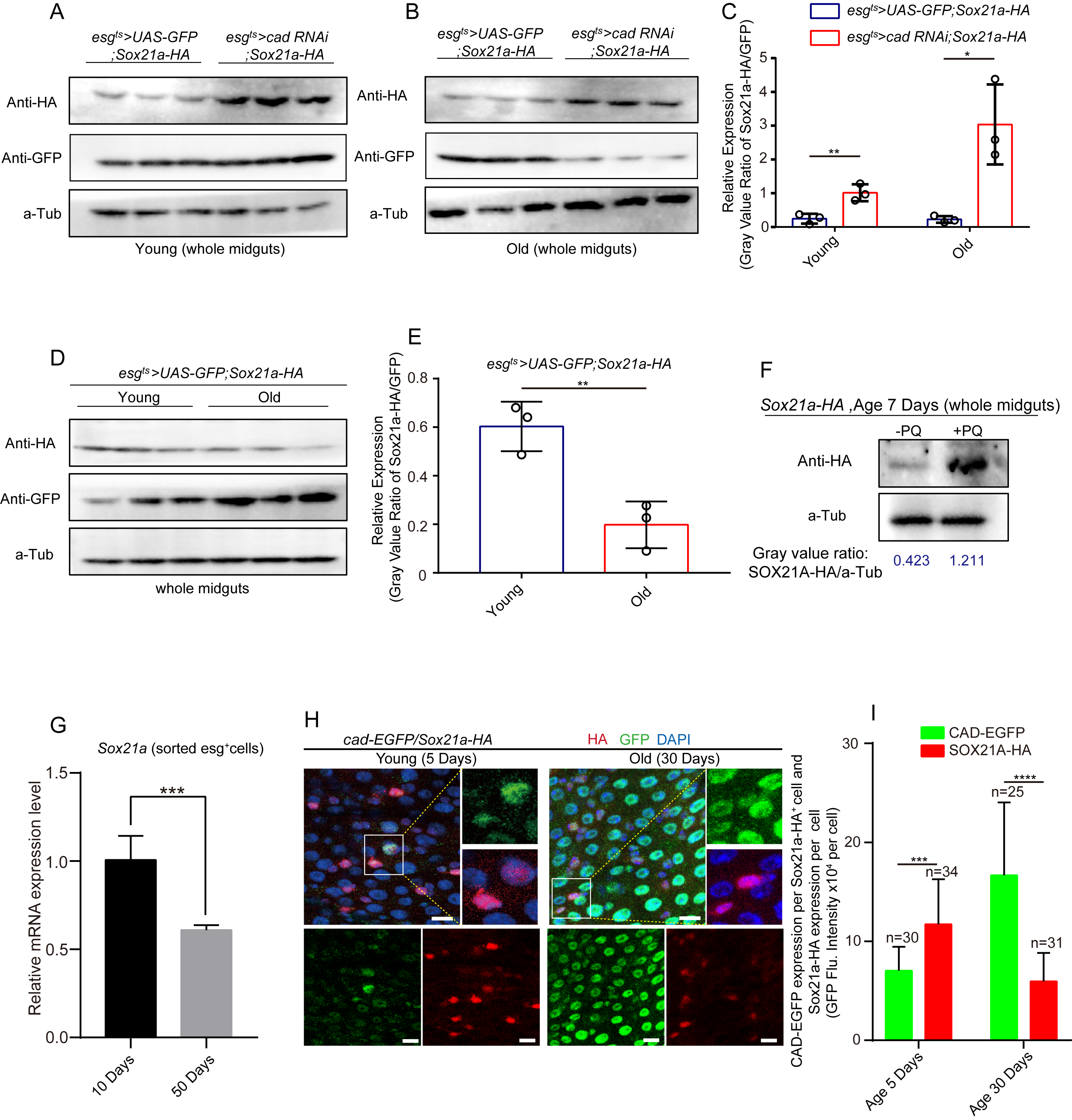

Supplement: S4 Fig — (A-B) Western blotting of SOX21A-HA of whole midguts from young (7 days; A) and old (35 days; B) control Drosophila (esgts-Gal4>UAS-GFP; Sox21a-HA) and Drosophila carrying esgts-Gal4>cad RNAi; Sox21a-HA. Loading controls, esg-GFP (GFP) and a-tubulin (a-Tub). (C) Quantification of the relative band intensity of SOX21A-HA to esg-GFP as shown in experiments (A-B). (D) Western blotting of SOX21A-HA of whole midguts from young (7 days) and old (35 days) control Drosophila (esgts-Gal4>UAS-GFP; Sox21a-HA). Loading controls, esg-GFP (GFP) and a-tubulin (a-Tub). (E) Quantification of the relative band intensity of SOX21A-HA to esg-GFP as shown in experiments (D). (F) Western blotting of SOX21A-HA of whole midguts carrying Sox21a-HA were treated with or without PQ (PQ-REC-1D). Loading controls, a-tubulin (a-Tub). (G) Relative mRNA fold changes of Sox21a in sorted esg+ cells from midguts of young (10 days) and old (50 days) Drosophila carrying esg-GFP and Sox21a-HA. The changes of expressions were plotted relative to the young Drosophila, which was set to 1. Error bars indicate the standard deviation (SD) of three independent experiments. (H) Immunofluorescence images of CAD-EGFP (green) and SOX21A-HA (red) staining with the midgut section from PMG of young (5 days) and old (30 days) Drosophila carrying cad-EGFP and Sox21a-HA. The separated channels of GFP and HA were indicated in the lower panel. The enlarged insets show Sox21a-HA+ cells (red) with CAD-GFP (green) staining. (I) Quantification of fluorescence intensity of CAD-EGFP and SOX21A-HA per HA+ cell as shown in (H). The number n is indicated. Each dot represents one SOX21A-HA+ cell. DAPI stained nuclei are shown in blue. Scale bars represent 10μm (S4H Fig). Error bars represent SD. Student’s t-tests were used to assess significance: *p < 0.05, **p < 0.01, ***p < 0.001, ****p < 0.0001, and NS (non-significant), which represents p > 0.05. (TIF) [file pgen.1009649.s004.tif]

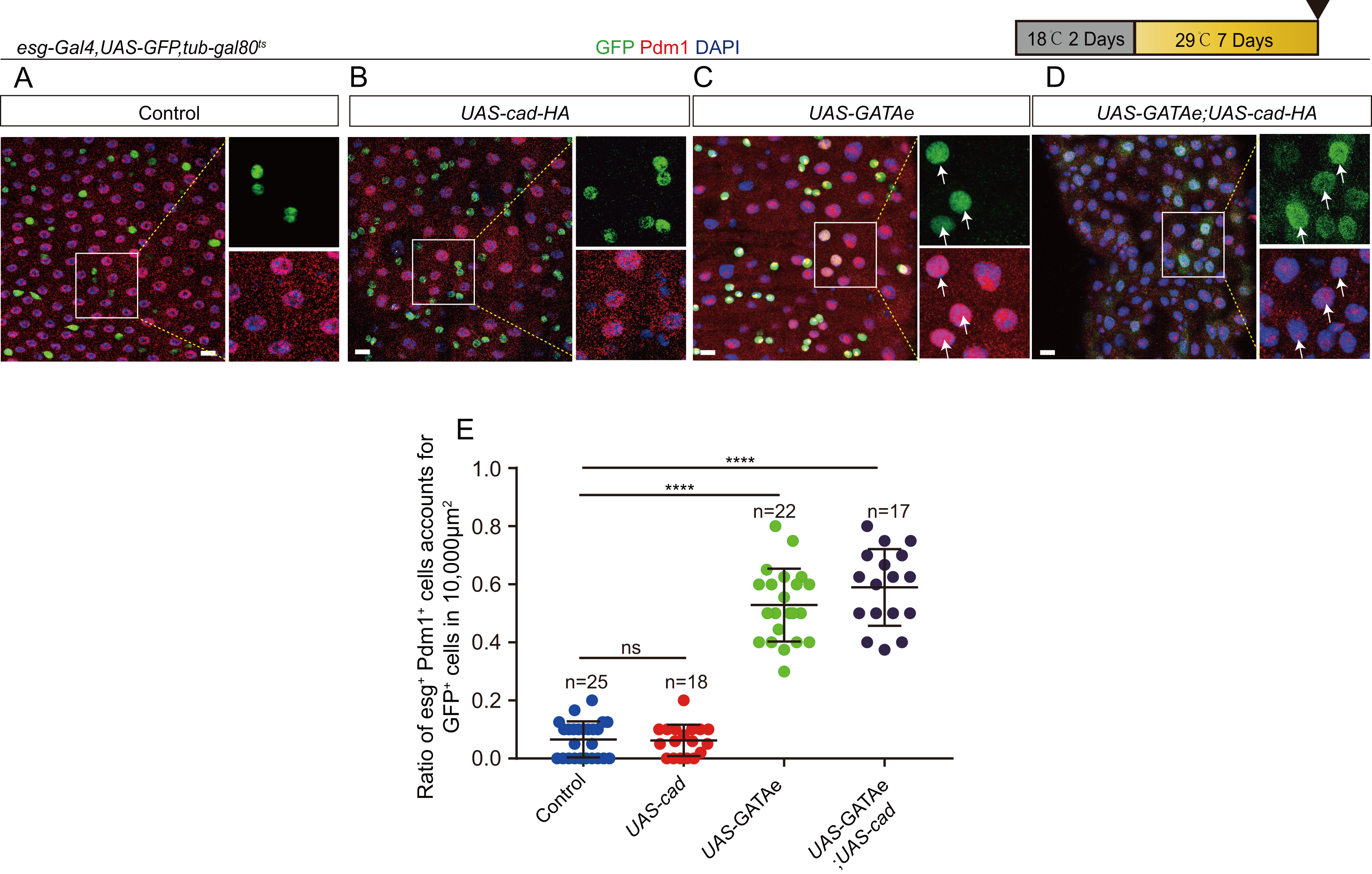

Supplement: S5 Fig — (A-D) Immunofluorescence images of esg-GFP (green) and Pdm1 (red) staining with the midgut in homeostasis. The midgut section from PMG of control Drosophila (A, esgts-Gal4>UAS-GFP), Drosophila carrying esgts-Gal4>UAS-cad-HA (B), Drosophila carrying esgts-Gal4>UAS-GATAe (C), and Drosophila carrying esgts-Gal4>UAS-cad-HA and UAS-GATAe (D). esg-GFP (green) represents ISCs and their differentiating cells. Pdm1 staining (red) was used to visualize differentiating ECs. White arrows indicate differentiating pre-ECs (esg-GFP+ and Pdm1+ cells). esg-GFP+ and Pdm1- cells are ISCs or EBs. esg-GFP- and Pdm1+ cells are mature ECs. (E) Quantification of the ratio of esg-GFP+ and Pdm1+ cells per 10,000 μm2 area of R4 region midguts of control Drosophila with genotypes as indicated in A-D. The number n represents counted ROI in midguts from each experiment. Each dot corresponds to one ROI (10,000 μm2 area). DAPI stained nuclei are shown in blue. Scale bars represent 10μm (S5A–S5D Fig). Error bars represent SD. Student’s t-tests were used to assess significance: *p < 0.05, **p < 0.01, ***p < 0.001, ****p < 0.0001, and NS (non-significant), which represents p > 0.05. (TIF) [file pgen.1009649.s005.tif]

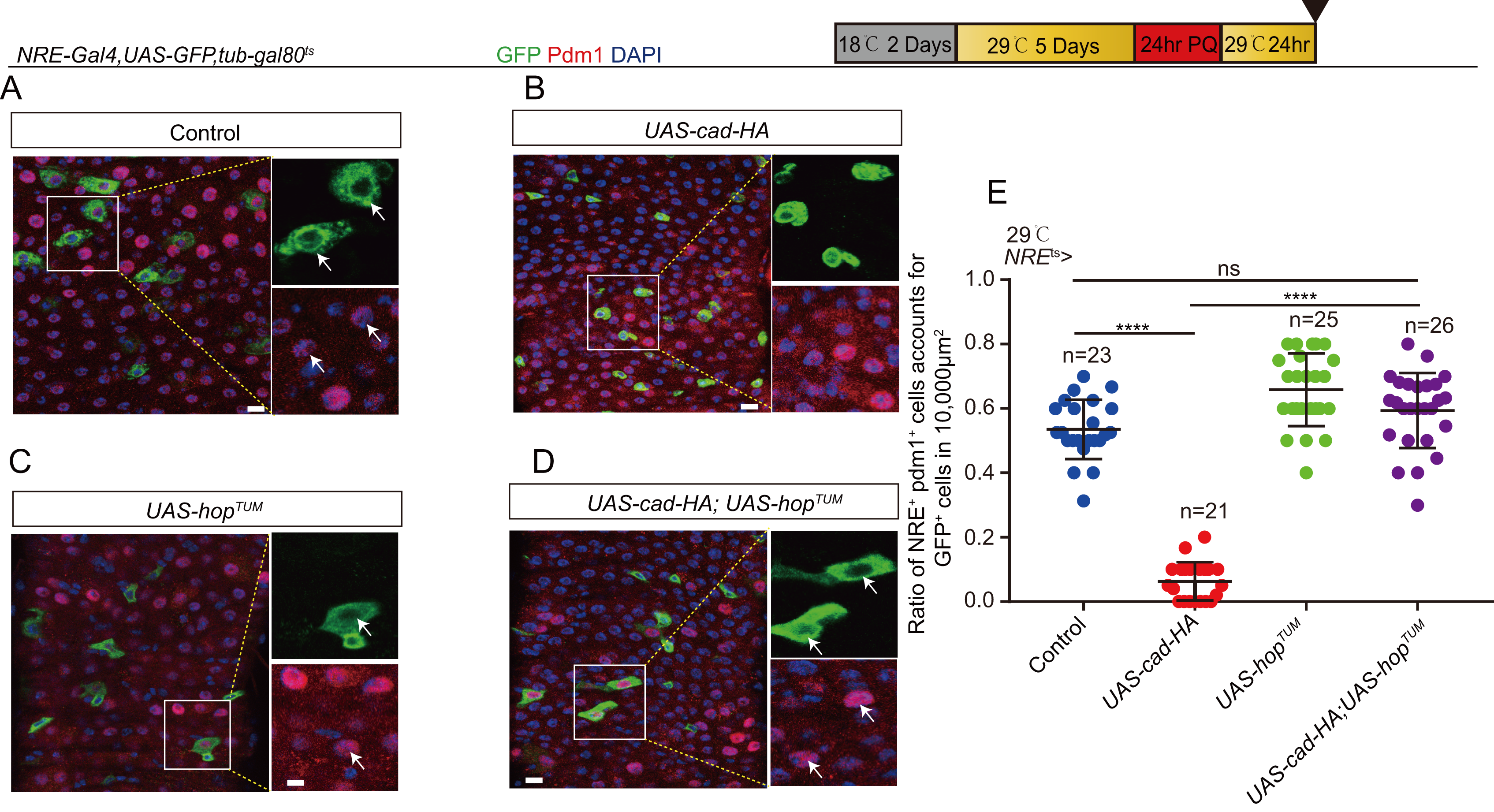

Supplement: S6 Fig — (A-D) Immunofluorescence images of NRE-GFP (green) and Pdm1 (red) staining with the midgut fed with PQ (PQ-REC-1D). The midgut section from the R4 region of control flies (A, NREts-Gal4-driven UAS-GFP), Drosophila carrying NREts-Gal4-driven UAS-cad-HA (B), Drosophila carrying NREts-Gal4-driven UAS-hopTUM (C), and Drosophila carrying NREts-Gal4-driven UAS-cad-HA and UAS-hopTUM (D). Pdm1 staining (red) was used to visualize differentiating ECs. White arrows indicate differentiating pre-ECs (NRE-GFP+ and Pdm1+ cells). NRE-GFP+ and Pdm1- cells are EBs. NRE-GFP- and Pdm1+ cells are matured ECs. (E) Quantification of the ratio of NRE-GFP+ and Pdm1+ cells Per 10,000 μm2 area of the R4 region midguts as shown in (A-D). The number n represents counted regions of interest in midguts from each experiment. Each dot corresponds to one region of interest (ROI = 10,000 μm2 area). DAPI stained nuclei are shown in blue. Scale bars represent 10μm (S6A–S6D Fig). Error bars represent SD. Student’s t-tests were used to assess significance: *p < 0.05, **p < 0.01, ***p < 0.001, ****p < 0.0001, and NS (non-significant), which represents p > 0.05. (TIF) [file pgen.1009649.s006.tif]

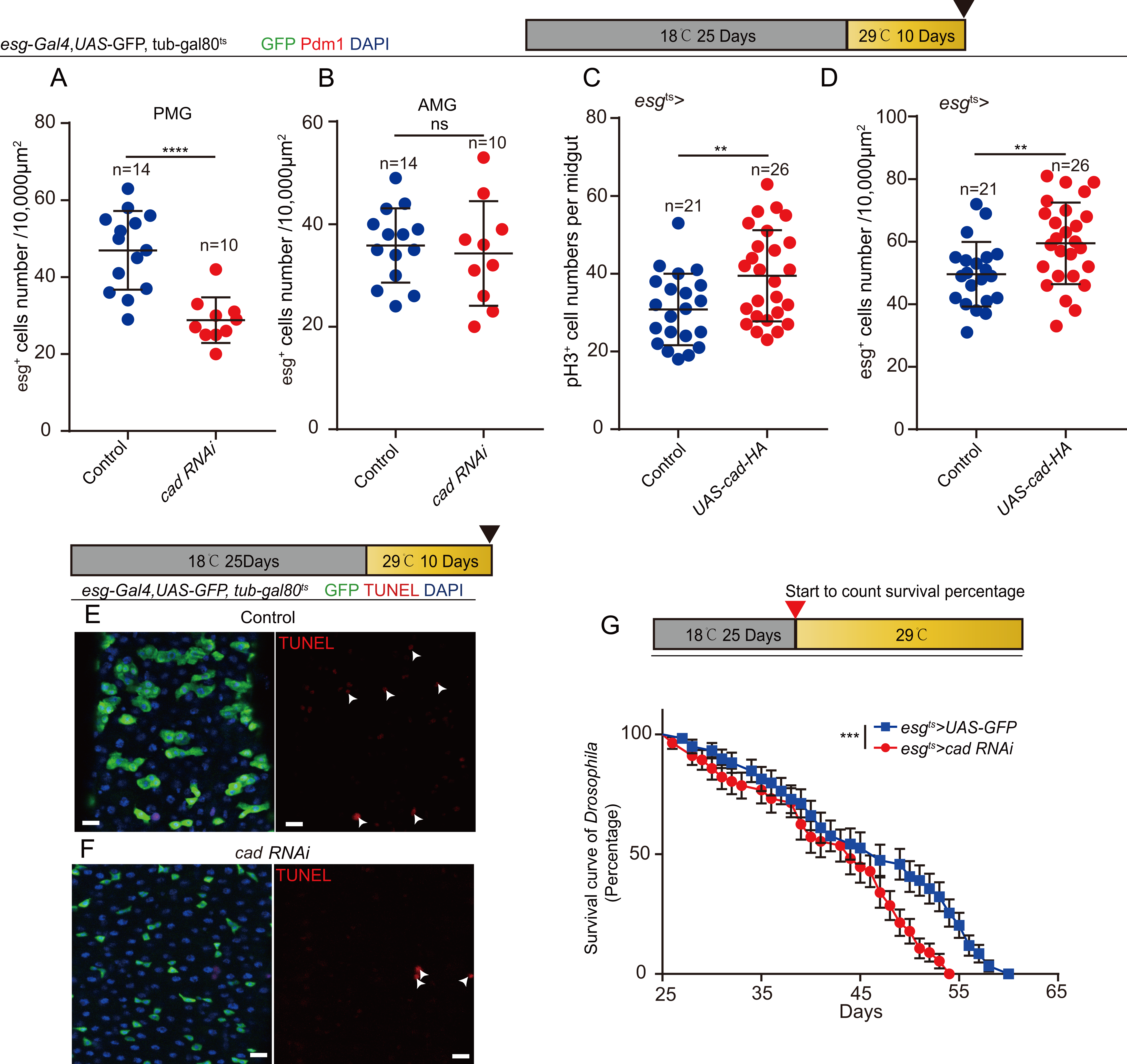

Supplement: S7 Fig — (A-B) Quantification of the esg+ cell number in AMG (B) or PMG (A) from 35-day-old (25 days at 18°C, then 10 days at 29°C) control Drosophila (esgts-Gal4>UAS-GFP) and Drosophila carrying esgts-Gal4>cad RNAi. The number n is indicated. Each dot corresponds to one ROI (10,000 μm2 area). (C-D) Quantification of pH3+ (C) or esg+ (D) cell numbers in midguts from 35-day-old (25 days at 18°C, then 10 days at 29°C) control Drosophila (esgts-Gal4>UAS-GFP) and Drosophila carrying esgts-Gal4>UAS-cad-HA. The number n represents the whole midguts in C. One dot corresponds to one midgut in C. The number n represents the ROI in midguts from each experiment in D. One dot corresponds to one ROI (10,000 μm2 area) in D. (E-F) Immunofluorescence images of esg-GFP (green) and TUNEL (red) staining with the midgut section from PMG of 35-day-old (25 days at 18°C, then 10 days at 29°C) control Drosophila (E, esgts-Gal4>UAS-GFP) and Drosophila carrying esgts-Gal4>cad RNAi (F). esg-GFP (green) identifies ISCs and their differentiating cells. TUNEL staining (red) was used to visualize the apoptotic cells. White arrowheads indicate apoptotic cells. (G) Survival rate of control Drosophila (esgts-Gal4-driven UAS-GFP) and Drosophila carrying esgts-Gal4-driven cad RNAi. Adult flies were cultured at 18°C for 25 days and shifted to grow at 29°C to turn on the UAS-Gal4 system. The survival rates of these flies were recorded on the 25th day. DAPI stained nuclei are shown in blue. Scale bars represent 10 μm (S7A, S7B, S7K and S7L Fig). Error bars represent SD. Student’s t-tests were used to assess significance. *p < 0.05, **p < 0.01, ***p < 0.001, ****p < 0.0001, and NS (non-significant), which represents p > 0.05. (TIF) [file pgen.1009649.s007.tif]
