# Supplementary material for: Aging-related upregulation of the homeobox gene caudal represses intestinal stem cell differentiation in Drosophila
Source: PLoS Genet. 2021 Jul 6;17(7):e1009649. doi: 10.1371/journal.pgen.1009649 (PMC8284806; doi:10.1371/journal.pgen.1009649)
Supplement: Attachment — Submitted filename: Response letter R1-9.docx [file pgen.1009649.s011.docx]

Dear Dr. Tang and Dr. Bosco,

Many thanks for handling our manuscript. All three reviewers have commented on the originality and importance of our findings. We appreciate their constructive suggestions. Below please find our point-by-point response (*italic maroon color)* to each of the comments (copied in full in black). We have also provided references cited at the end of the response.

We hope that our revision is satisfactory.

Best wishes!

Haiyang Chen

(On behalf of all authors)

**Reviewer comments:

Reviewer #1:**

In this manuscript, Wu and colleagues addressed the molecular mechanisms that underlie age-related decline in stem cell differentiation efficiency in Drosophila. Using the available genetic tool kits, authors identified the function of caudal (cad), a homeobox transcription factor in the differentiation of midgut intestinal stem cells (ISCs) to enterocytes (EC). Increased cad expression in ISCs and enteroblasts (EB) of aged flies prevents differentiation of progenitor cells by inhibiting the JAK/STAT-SOX21a-GATAe signaling pathway. Cell-specific knockdown of cad in the midgut ISCs and EBs increases differentiation efficiency, thereby restrain age-related hyperplasia in Drosophila.

Of fundamental interest, this study is important and will be of interest to broader readers of PLOS Genetics. I have a few suggestions and comments below that may be potentially useful for the authors to consider:

*Response:
We appreciate that this reviewer finds our study original and important. We believe the revisions we made as detailed below have addressed the concerns raised.*

Major comments:

1. By employing a conditional knockdown approach, the authors present a key find that the reduction of cad in ISCs and progenitor cells increases differentiation of ISC to EC (Figure 2).
a. In these experiments, the authors used esgts-Gal4 to drive two reporter lines (UAS-GFP and UAS cad-RNAi). The authors should either report the RNAi-mediated knockdown efficiency or cite the previous references for the UAS cad-RNAi.
b. Lucidly report the conditional knockdown protocol in the methods sections. In methods, ‘The tub-Gal80ts transgenes Drosophila were grown at 18 ℃ and transferred to 29 ℃ for 7 days, followed by dissection’ and in figure legend 2, ‘….the midgut section from the R4 region of 9-day-old (2 days at 18 ℃, then 7 days at 29 ℃) control Drosophila….’

*Response:*

*We appreciate these suggestions.*

*a. In the revised manuscript, we have included the data that show the RNAi-mediated knockdown efficiency of cad of these two RNAi lines (UAS-cad RNAi^BL57546^ and UAS-cad RNAi^BL34702^) by RT-qPCR. This data showed a significant reduction of cad mRNA expression in the midguts of flies carrying either tub-Gal4>UAS-cad RNAi^BL57546^ or tub-Gal4>UAS-cad RNAi^BL34702^ compared to that in the midguts of flies carrying tub-Gal4>UAS-GFP (S2A Fig).*

*b. We apologize that we were not clear enough in description the conditional knockdown protocol in the methods and legends. To avoid redundance and make figure legends concise, we have described the conditional knockdown protocol clearly in the revised methods (sub-titled by “Drosophila breeding and maintenance”) and depleted the redundance description in the figure legends.*

2. Pdm1 staining used to visualize differentiating ECs is inconsistent with high background signals. Do the authors have any comments? Also, the authors should include in detail methods for image acquisition, processing, and fluorescent intensity quantification.

*Response:*

*Thanks for this question. Indeed, the Pdm1 staining showed high background signals. We guess it is because of the bad quality of Pdm1 antibody (unfortunately, there is no better Pdm1 antibody up to now). In our experiments, Pdm1 (a* *nuclear-localized protein) staining was just used as a marker to label the ECs. We didn’t quantify the fluorescent intensity of images with Pdm1 staining. Thus, we think the high background signals of Pdm1 staining do not affect the conclusion.* *In the revised manuscript, we have included more detail of the methods for image acquisition, processing, and fluorescent intensity quantification.*

3. It is interesting that esgts-Gal4-driven UAS cad-RNAi in the progenitor cells reduces gut hyperplasia in aged flies.
a. It would be useful to report the expression of JAK/STAT signaling molecules in the midgut of aged esgts-Gal4 > UAS cad-RNAi flies.
b. Does depletion of downstream signaling molecules JAK/STAT-SOX21a-GATAe reverts the midgut phenotype of esgts-Gal4 > UAS cad-RNAi in aged flies?

*Response:*

*a. We appreciate this suggestion. We have checked the activity of the JAK/STAT signaling in the midgut of aged esg^ts^-Gal4 > UAS cad-RNAi flies as indicated by the mRNA expression of the STAT downstream target Socs36E (Fig. 7L ). All these data indicated that cad functions as the upstream to repress the activation of JAK/STAT signaling.*

*b. Thanks for this question. We have checked the genetic interaction between cad and the JAK/STAT-SOX21a-GATAe signaling in the midguts of aged flies. The midgut phenotype of aged flies carrying esg^ts^-Gal4 > UAS cad-RNAi was partially reversed by depletion of either Sox21a or Stet92E (See Fig. 7M-7P in the revised manuscript)*

4. Some comments on the following sections for authors to consider:
a. In the methods section, more details should be provided and alternatively, if the procedures are previously described, then the reference citations should be included.
b. Reference citations for the genetic tools used in the manuscript are missing.
c. Authors should make sure that the genotypes are clear, correct, and consistent throughout the manuscript.
d. Figure legends are too wordy and lengthy - consider reducing the repetitive statements.

*Response:*

*We apologize for not clarifying the methods and genotypes clearly and appreciate these suggestions.*

*a. In the revised manuscript, we have provided more details in methods section and included the relevant reference citations.*

*b. The relevant reference citations for the genetic tools have been included in the revised manuscript.*

*c. We have checked and modified the Drosophila genotypes throughout the revised manuscript. In addition, all Drosophila lines used in this study are listed in Supplementary S3Table.*

*d. We have edited the figure legends to make them clear and concise in the revised manuscript.*

5. The conclusion that the increased cad expression in the progenitor cells represses age-associated ISCs differentiation in flies is well established. Discuss what and how cad expressions regulated in young and aged flies would be useful. Also, how does the decline in differentiation efficiency of the midgut cells affect the interorgan communication in flies?

*Response:*

*We appreciate this suggestion. In our revised manuscript, we have discussed what and how cad expressions regulated in young and aged flies. The possible effects of the decline in differentiation efficiency of the midgut cells affect the interorgan communication in flies were also discussed.*

Minor comments:
1. Drosophila is misspelled as Drosospila in several places.

*Response:*

*We apologize for these clerical errors and have corrected them in the revised manuscript.*

2. Methods section corresponding to figure 1B is missing.

*Response:*

*The protocols of FACS and RT-qPCR have been included in the revised methods section.*

3. Methods section should include fly culturing and maintenance for the aging experiments.

*Response:*

*We have included fly culturing and maintenance for the aging experiments in the methods section of the revised manuscript.*

4. Page 9, ‘….midguts of wild-type control flies’ should be ‘…..midguts of experimental/genotype control flies’

*Response:*

*Thanks. We have corrected this mistake in the revised manuscript.*

5. Page 34, repetition of the words: ‘White arrows indicate differentiating pre-ECs (both esg-GFP+ and Pdm1+ (esg-GFP+ and Pdm1+) cells)’

*Response:*

*We have deleted this repetition in the revised manuscript.*

6. Figure 4A legend: ‘esgts-Gal4-driven UAS-GFP’ – according to methods and figure, reporter line is UAS-LacZ.

*Response:*

*We have corrected this mistake.*

7. Figure 4C: ‘SOX21A-HA; cad RNAi’ should be ‘cad RNAi; SOX21A-HA’

*Response:*

*Thanks! We have corrected this mistake.*

8. Figure 6C: ‘UAS-cad-HA; 10xSTAT-GFP’ should be ‘10xSTAT-GFP; UAS-cad-HA’

*Response:*

*Thanks! We have corrected this mistake.*

9. Figure 7 legend: esgts-Gal4-driven cad-RNAi was induced from 26 day after fly eclosion, not at.

*Response:*

*Thanks! We have corrected this mistake.*

Reviewer #2:

In this study, Wu and colleagues investigated the role of caudal (cad) in the differentiation of intestinal stem cells in Drosophila midguts. The authors identified that cad, a homeobox transcription factor, is upregulated in aged flies. Knockdown of cad in intestinal stem/progenitor cells promotes their differentiation towards enterocytes, whereas overexpression of cad prevents differentiation and regeneration upon injury. The author further determined that cad regulates ISC differentiation through Jak/STAT-Sox21a-GATAe signaling cascades. Lastly, the authors provide evidence suggesting reduction of cad in intestinal stem/progenitor cells prevent gut hyperplasia in aged Drosophila. Overall, this study is well-designed and the evidence strongly support the important role of cad in the regulation of ISC/progenitor cell differentiation. However, given the title and the beginning of the current manuscript, I expected to see data regarding the role of cad in both young and aged flies. It turns out a major portion of this study is focused on the function of cad in homeostasis or regeneration. Below are some major issues I believe the authors should address.

*Response:*

*We thank this reviewer for appreciating the importance of our findings. Thanks for these suggestions which indeed help improve our manuscript. We believe the revisions we made as detailed below have addressed the concerns raised and further strengthened our conclusions*

1. The authors first examined the expression levels of CAD-GFP in young and old flies. It seems to me that CAD is expressed in all cell types, perhaps at a higher level in ECs than in ISCs (Fig.1C). Based on the quantification of CAD-GFP intensity, the levels of CAD increase in all cell types upon aging. I believe the authors should clarify the expression pattern of CAD.

*Response:*

*We agree with this reviewer. Indeed, CAD is expressed in all cell types of midguts.* *In this study, we found that the expression of CAD in some ECs and EEs is indeed higher than it in ISCs of young flies. And the levels of CAD increased in all cell types of posterior midguts upon aging. We agree with this reviewer and have clarified this expression pattern of CAD in the revised manuscript. In this study, our data emphasize that the expression of CAD in ISCs/EBs of aged flies increases more dramatically than that in ECs and EEs, which suggests that CAD may have an important function in ISC aging.*

2. Is there antibody available to detect endogenous CAD? If so, it would be nice to show endogenous CAD levels increase in aged animals. If not, the authors should at least examine the expression of CAD-GFP in young and aged flies by western blot, which could support their claim using a different method.

*Response:*

*We appreciate this suggestion. Unfortunately, up to now, there is no available commercial CAD antibody. We had tried to generate CAD antibody,* *yet we failed. As this reviewer suggested, we detected the expression of CAD-GFP in midguts of young and aged flies by western blot (see S1F Fig in the revised manuscript).*

3. Does depletion of cad in ISCs have an effect on their proliferation? The phenotype looks like a block of differentiation, but it would be important to rule out some other possibilities. Therefore, I suggest the authors should examine the proliferation rate of ISCs by phospho-Histone H3 staining.

*Response:*

*We appreciate this suggestion. In the revised manuscript, we have detected the proliferation rate of ISCs by quantifying the phospho-Histone H3 positive (pH3^+^) cells in midguts with CAD depleted. The result suggested that the proliferation rate of ISCs has no significant difference between control and depletion of cad in esg+ cells (Fig. 2L). However, the cell number of esg+ cell nest increased slightly in the posterior midguts of flies carrying esgts>cad RNAi (Fig. 2E).*

4. Many of the functional experiments were done using RNAi-mediated knockdown, except Fig3J-M. Have the authors compare the effect of cad knockdown versus knockout?

*Response:*

*Thanks for this question. Since cad-null (cad^2^/ DF (2L) Exel7079) mutant flies died before eclosion, it is impossible for us to observe the adult midguts of cad-null flies.* *Alternatively, we generated cad-null MARCM clones to detect functions of cad in regulating ISC differentiation (see Fig 3J-3S, and S3M-S3O Fig). By analyzing the cad-null MARCM clones, we got the same conclusion that cad prevents ISC-to-EC differentiation.*

5. The experiments of examine CAD function in ISC/progenitor cells were all done in young flies. Have the authors perform similar experiments in aged animals?

*Response:*

*We appreciate this suggestion. In the original manuscript, we performed the Delta staining and counting esg+ cell number in the esg^ts^-Gal4 > UAS cad-RNAi flies upon aging (See Fig 7I-7K and 7D-7F in the revised manuscript). In the revised manuscript, we have performed more experiments in aged animals to examine CAD function in ISC/progenitor cells, including Pdm1, pH3, and TUNEL analyses. Please see these data in Fig 7A-7C, 7G-7H, S7A-S7D, and S7E-S7F in the revised manuscript.*

6. The authors mentioned in the beginning that cad expression in ISCs/EBs can be induced by bacteria infection. Similarly, does PQ-induced injury affect CAD expression levels in flies midguts?

*Response:*

*Thanks for this question. In the revised manuscript, we have detected the expression of CAD in midguts of flies after PQ-induced injury by staining the endogenous CAD-EGFP and quantifying their fluorescence intensity (see S1J-K Fig). Indeed, PQ-induced injury promoted the expression of CAD in the posterior midguts of flies. This result is consistent with a previous study from Choi YJ and colleagues in 2008 [1].*

*.*

7. The authors showed that overexpression of cad in ISC/progenitor cells prevent their differentiation to ECs. Are those ISC/progenitor cells able to proliferate upon injury? Also, is there an effect on the overall fitness of flies overexpressing cad in ISC/progenitors?

*Response:*

*We appreciate these questions and have performed additional analyses. After PQ-induced injury overexpression of CAD in ISCs/progenitor cells of flies induced a significant increase of ISC proliferation rate indicated by pH3 staining compared to the control (see Fig 2M in the revised manuscript). This may because that overexpression of CAD in ISCs/progenitor cells caused the failure of ISC-to-EC differentiation, which in turn caused the formation of EB tumors in midguts. The EB tumors send EGF(Spi) and JAK-STAT ligands (Upd2) to neighboring ISCs to drive their proliferation (Chen et al., 2016; Zhai et al., 2015)[2, 3].*

*Moreover, overexpression of CAD in ISCs/progenitor cells caused an increase of mortality of flies under low dose PQ-induced injury compared to the control flies (see Fig 7Q in the revised manuscript). This data suggested that overexpression of CAD in ISCs/progenitor cells reduces the overall fitness of flies.*

8. The authors examined SOX21A-HA expression levels upon cad depletion by immunofluorescence staining. I would help their claim if the authors can show the increase of protein levels by western blot. Also, it would be interesting to compare the SOX21 levels in young versus old flies.

*Response:*

*We appreciate this suggestion. In the revised manuscript, we have shown the increase of protein levels of SOX21A-HA in esg+ cells of flies with cad depletion by western blot (S4A-S4C Fig). Moreover, we have compared the SOX21 levels in young versus old flies by western blot (S4D-S4E Fig), RT-qPCR (S4G Fig), and immunostaining (S4H-S4I Fig). These data showed that SOX21a decreased in midguts upon aging.*

9. It is not clear to me why the authors decided to investigate the interaction between GATAe and cad. Is GATAe one of the differentially expressed genes in their RNA-seq results? Given GATAe is downstream of Sox21a, one would naturally think GATAe is downstream of cad.

*Response:*

*Thanks for this question. We agree with this reviewer that GATAe, indeed, is a demonstrated downstream of Sox21a to regulate ISC-to-EC differentiation [4, 5]. We detected the genetic interaction between GATAe and cad in this study is just because we wanted to further confirm that cad regulates ISC-to-EC differentiation through the JAK/STAT-SOX21A-GATAe signaling.*

10. The authors showed knockdown of cad in ISC/progenitor cells prevent gut hyperplasia in aged flies. Does that have an effect on the overall fitness of aged flies or their longevity?

*Response:*

*We appreciate this suggestion.* *We have analyzed the lifespan of flies carrying esg^ts^>UAS cad RNAi (flies were cultured at 18℃ for 25 days, then these flies were transferred to grow at 29℃ to induce cad RNAi). However, we didn’t find a significant increase (but a minor reduction) in the longevity of flies with cad depletion compared to the control flies (esg^ts^>UAS-GFP) (see S7G Fig in the revised manuscript). The possible reason that knockdown cad does not increase fly lifespan maybe because that depletion of cad causes over-reduction of ISCs under normal conditions or cad has other unknown functions in ISCs/progenitor cells.*

Reviewer #3:

The report by Wu and colleagues investigates the role of the transcription factor Caudal (CAD) in the control of the Drosophila midgut intestinal lineage. The authors first confirm previous finding indicating that cad is upregulated in the gut of aged flies. Then, using a protein trap they show that although CAD protein expression increases in every cell type of the gut upon aging there is a greater upregulation in ISCs and EBs. The authors further show that inactivation of cad in EBs leads to the accumulation of cells expressing the EC marker, Pdm1, in normal condition of homeostasis while overexpression of cad decreases EC production after gut injury. Using classical Drosophila genetic tools and RNASeq, the authors bring some evidences suggesting that caudal regulates EB differentiation in EC upstream of the well described genetic framework composed of JAK-STAT/Sox21a/GATAe. Lastly, the authors find that a depletion of cad in the progenitor cells (ISC - EBs) leads to a decrease in hyperplasia formation in aged guts possibly because of the role of cad in the decline of ISC capacity to differentiate over time.

While a role for cad in midgut innate immune response and in ISC proliferation in midgut was already reported, its function in EBs to regulate differentiation and ECs formation was unexplored. Overall, the paper is clear and the conclusions are supported by quantified experiments. In my opinion, the weakness of the study at this stage is that the authors fail to completely demonstrate that the role of cad is restricted to EB-EC differentiation. Further work should discriminate between a role in the control of stem cell proliferation as reported in Choi et al 2008 and a role in EC differentiation upon homeostatic condition and after injury. The proposed model itself is quite convincing in normal condition of homeostasis (although not free of alternative explanations) but it is rather preliminary to extend it to aged guts and hyperplasia formation. Indeed, the data in old guts are limited to a partial phenotypical characterization of cad depletion in progenitor cells.

*Response:*

*We thank this reviewer for appreciating the novelty of our findings. Thanks very much for these comments and suggestions which indeed help improve our manuscript. We believe the revisions we made as detailed below have addressed the concerns raised and further strengthened our conclusions.*

Here are some issues the authors should address before publication can be recommended:

Major Points:

1. The authors report that in the RNA-seq from Duta et al, cad is highly expressed in ISC and EB in regions R4-R5 but omitted to mention that cad is also highly expressed in EC and even more in EE in this region under normal condition of homeostasis.

*Response:*

*We appreciate this reviewer to point our omissions. Indeed, CAD is expressed in all cell types of midguts. In the revised manuscript, we have provided the expression of cad in EC and EE in the RNA-seq from Duta et al (see S1D Fig )[6]. In this study, we also showed that the expression of CAD (indicated by endogenous CAD-EGFP reporter) in some ECs and EEs is indeed higher than it expressed in ISCs and EBs of young flies (see Fig 1E and Fig1G). We agree with this reviewer and have clarified the expression pattern of CAD in the revised manuscript.*

2. Regarding CAD-EGFP expression in the different cell types of the midgut in Fig1: the authors limit their analysis to the region R4 in Fig1 C-N. It would be informative to report CAD-EGFP expression in the other regions of young and old guts. Is there any obvious difference as suggested by the RNA-seq data or is CAD uniformly distributed along the gut?

*Response:*

*We appreciate this suggestion. In the revised manuscript, we have included the CAD-EGFP expression in the other regions (R1-3 and R5) of midguts of young and old flies (S1G-S1I Fig). The data of CAD-EGFP expression in this study were consistent with the RNA-seq data from Duta et al[6] (also see S1B-S1D Fig). Both our CAD-EGFP staining and the RNA-seq data from Duta et al showed that CAD does not uniformly distribute along the gut. Both data indicate that CAD mainly expresses in the posterior (R4 and R5 regions) midgut but barely or slightly expresses in the anterior (R1 and R2 regions) and CCR (R3 region) midguts.*

The authors propose that CAD modulates sox21a expression. sox21a expression has been reported in Zhai et al 2017 to be higher in ISC than in EB and to be totally shut down before the Notch activity reporter in the EBs. Here, looking at Fig 1E and 1H CAD-GFP seems to be less upregulated in ISCs (Dl+ cells) than in ISC/EB (esg+ cells) in general. It would be interesting to confirm that indeed CAD is differentially expressed between ISC and EB by co-staining of esg-lacZ/Dl/CADGFP or Dl/NRElacZ/CADGFP. Alternatively, it would be pertinent to compare Sox21a-HA and CAD-GFP expression profile in ISC/EBs of young and old guts. Is there a transient upregulation of CAD at the same time as Sox21 disappearance?

*Response:*

*We appreciate this suggestion. We agree with this reviewer that it is important to check the differential expression of CAD between ISCs and EBs by co-staining of esg-lacZ/Dl/CADGFP. Our data showed that the EBs (esg+Dl- cells) usually displayed a higher CAD-GFP expression level than the ISCs in the same ISC-EB nests during homeostasis (see Fig 1M-1N in the revised manuscript). Since we proposed that cad function as an upstream to prevent the expression of sox21a, it is reasonable to see that cad expresses higher in EBs than in ISCs and sox21a expresses lower in EBs than in ISCs as reported by Zhai et al 2017[4].*

*Moreover, we have compared Sox21a-HA and CAD-GFP expression profiles in ISC/EBs of young and old guts by co-staining of CAD-GFP and Sox21a-HA. It indeed showed that when CAD-GFP increased in old ISC/EBs the expression of Sox21A-HA downregulated at the same time (see S4H-S4I Fig in the revised manuscript).*

3. One of the main conclusions of the authors is the role of cad in the regulation of EB differentiation. I feel that the data backing up this conclusion are rather weak. Indeed, it is currently mainly based on a partial characterization of cad knockdown or overexpression in progenitor cells. This phenotypical characterization needs to be completed to rule out other hypothesis than a role of cad in EB differentiation.
It is essential for each condition to look at ISC proliferation using PH3 staining. This is important to rule out the possibility of an increase of ISC division leading to an excess of pre-EC production, expressing Pdm1 while maintaining some GFP due to protein perdurance. A decrease of ISC proliferation or ISC numbers could also explain a reduction of EC production upon overexpression of cad instead of a differentiation defect. This is also important since caudal has been proposed to regulate cell proliferation in the adult posterior midgut by Choi et al 2008.
Quantifications of the number of ISC (Dl+ cells) and esg+ cells are reported only for esgts>cad RNAi, and is limited to a ratio of Pdm1+ GFP+ / total GFP+ cells for the other conditions. In order to be able to properly interpret the results it would be important to have for each conditions de number of PH3+ cells, the number of Dl+ cells, and total GFP+ cells per region or per guts.
CAD appears to be enriched in regions R4 and R5 but is expressed in all the regions of the guts. The phenotypes reported here only mention R4 region. What about the other regions? Sox 21a appears to have different functions on ISC proliferation and progenitor differentiation in the anterior midgut or in the posterior midgut, is it true also for CAD?

*Response:*

*We appreciate these comments and suggestions. We agree with this reviewer that it is important to rule out the possibility of an increase of ISC division leading to an excess of pre-EC production by looking at ISC proliferation using pH3 staining for each condition. We have performed these experiments and found that the proliferation rate of ISCs (indicated by pH3 staining) has no significant difference between control flies and flies with cad depleted in esg+ cells under homeostasis condition (see Fig 2L in the revised manuscript). Similar to the Sox21a mutant, overexpression of cad in esg+ cells led to a slight increase of the ISC proliferation rate in midgut as indicated by pH3 staining (see Fig. 2L in the revised manuscript). The slight increase of ISC proliferation (indicated by pH3 staining) in cad-overexpressed midguts (see Fig. 2L) may because that overexpression of CAD in ISCs/progenitor cells caused the failure of ISC-to-EC differentiation, which in turn caused the formation of EB tumors in midguts. The EB tumors send EGF(Spi) and JAK-STAT ligands (Upd2) to neighboring ISCs to drive their proliferation (Chen, et al., 2016; Zhai, et al., 2015)[2, 3]. According to the suggestion of this reviewer, we have quantified the numbers of Dl+ cells and esg-GFP+ cells per anterior and posterior midguts of flies with cad depletion or overexpression under* *homeostasis (See S2E-S2H Fig) and after injury (see S2I-S2L Fig in the revised manuscript).*

*We have also analyzed the numbers of esg+ cells and Dl+ cells of the anterior midguts of flies with cad depleted. Unlike sox21a mutant flies that have an obvious phenotype in both the anterior midguts and in the posterior midguts, we found that depletion of cad in the anterior midguts did not show obvious phenotype (see Fig.S2B-S2D, S2E, S2G, S2I, and S2K). We guess that the possible reason is that the expression of CAD is almost restricted specifically in the adult posterior midgut (according to Choi et al 2008 [1] and our CAD-EGFP reporter staining* *as shown in S1H Fig). However, we indeed found that ectopic overexpression of CAD in the anterior midgut led to an increase of esg+ cells and Dl+ cells (Fig. S2E and S2G). These data suggest that cad may only function as an upstream of Sox21a in the posterior midgut but not in the anterior midgut under physiological conditions.*

4. The authors proposed that cad knockdown phenotypes are reminiscent of sox21a overexpression. In Zhai et al., 2015 sox21a overexpression leads to EB differentiation into EC but this is associated with a decrease in progenitor cell number (esg+ cells). This is different here, esg+ cell number remains the same upon cad RNAi expression. This is the number of ISCs (Dl+ cells) that decrease. The authors do not comment on this major difference. This should be discussed. This is not clear how after 7 days of cad knockdown a decrease of ISCs is observed but the number of GFP+ cells remains constant. Since the ISCs are the only dividing cell type in the midgut, how the same number of GFP+ cells can be produced while stem cells are lost? Is there an increase in the proliferation of the remaining ISC in order to compensate?
The authors do not address general cell death in these conditions. Is the ISC loss (Dl+ cells) due to differentiation as proposed by the authors or is it related to cell death? This could be tested both searching for increase ISC cell death in mutant tissue, and test a potential rescue in conditions preventing cell death.

*Response:*

*We appreciate these questions and suggestions.* *We think the different* *phenotypes between* *cad knockdown and sox21a overexpression that this reviewer mentioned above may because the SOX21A expression level promotes by cad knockdown is less than the SOX21A expression level that induces by esg-Gal4-driven sox21a cDNA overexpression in Zhai et al., 2015[3]. Thus, the phenotypes of cad knockdown (esg-Gal4-driven UAS-cad RNAi) are relatively weaker than those of sox21a overexpression (esg-Gal4-driven UAS-sox21a cDNA). Another possible reason is that sox21a maybe not the only downstream of cad to regulate ISC differentiation. The other undetected downstream of cad make the phenotypes of midguts with cad knockdown are not fully the same as those seen in midguts with sox21a overexpression in Zhai et al., 2015[3]. In the revised manuscript, we have included this discussion.*

*To explain the phenotype why after 7 days of cad knockdown a decrease of ISCs is observed but the number of esg-GFP+ cells remains constant, we think the possible explanation is that, although the number of Dl+ ISCs (they are esg-GFP+ cells) reduces the number of the differentiating pre-ECs (they are esg-GFP+ cells；they are from the differentiated ISCs; it takes time for these differentiating pre-ECs to became matured ECs( esg-GFP- cells)) increases in cad-depleted midguts, which in turn leads to the number of esg-GFP+ cells remains constant.*

*We have checked the ISC proliferation rate (as indicated by pH3 staining) of midguts with cad knockdown. We did not see a significant increase in the proliferation rate of the remaining ISCs in cad-depleted midguts (see Fig. 2L). We have checked the general cell death of cad-depleted midguts by performing TUNEL staining analyses. We did not observe a significant increase in apoptotic cells in cad-knockdown midguts compared to the control (see Fig. S2M-S2N). Moreover, blocking cell death by inducing rpr RNAi could not reverse the phenotype of ISC reduction seen in cad-knockdown midguts (see S2O-S2R Fig). Thus, we got the conclusion that ISC (Dl+ cells) loss in cad-knockdown midguts is due to ISC-to-EB differentiation but not results in cell death.*

5. The authors limit their clonal analysis of cad RNAi and cad overexpression phenotypes to a ratio of Pdm1+ cells. To properly interpret the data, it would be important to know at least how many cells per clone are found in each condition (indication of proliferation). Alone the ratio of Pdm1 + cell per clone is not very meaningful, a change in the number of Pdm1+ cells could be due to an increase or a decrease of ISC proliferation and not to a change in differentiation capacity. In Fig3L, clones seem much smaller upon overexpression of cad compared to control. Is the decrease of Pdm1+ cells due to less proliferation rather than a defect of differentiation? It would also be essential to have a better idea of the composition of the clones. Dl+ cells (number of ISC per clone), NRE lacZ to have an idea of EB accumulation and prospero+ cells to have the number of EE cell per clone. This is especially important since the depletion of sox21a (the proposed main downstream effector of cad) affects not only EC differentiation but also ISC proliferation and EE prospero + cells production.

*Response:*

*We appreciate these comments and suggestions. We have included additional analyses in the revised manuscript.*

1) *We have quantified cell number per clone in each condition (see Fig.3N). The data indicated that depletion of cad did not affect ISC proliferation, while overexpression of cad reduces ISC proliferation (this phenotype is similar to the sox21a mutant clones reported by Zhai et al 2015)[3].*
2) *We also used different cell markers (including Dl, Pdm1, and Prospero staining) to analyze the composition of the cad-null and cad-overexpression clones (see Fig.3J-3N, Fig.3O-3R, and Fig.S3M-S3N).*
3) *The cells in* *cad-overexpression clones (see Fig.3L) were all diploid small cells without Pdm1 staining. By performing additional EE staining experiments,* *we found that these cells in cad-overexpressing clones also did not express the enteroendocrine cell marker Prospero (see Fig. S3O). These phenotypes of the cad-overexpressing clones are also consistent with the sox21a mutant clones reported by Zhai et al 2015[3].*
4) *We also performed Dl staining to label ISCs in MARCM clones (see Fig. 3O-3R).* *Cad-overexpression clones exhibited a significant low ratio of DI+ cells compared to the control clones.*
5) *Since we only have flies carrying NER-lacZ elements on chromatin X, we failed to generate flies with both cad MARCM clones and NER-lacZ reporter.*

*Since cells in cad-overexpression clones are all Pdm1 negative and Prospero negative and most of them are also Dl negative, we got the conclusion that these cells in the cad-overexpression clones are most EBs.*

*According to the above analyses, cad-overexpression clones and sox21a-mutant clones share quite similar phenotypes.*

6. Zhai et al., 2017 reported that esgts> sox21a guts form nest containing a slightly increase in the number of cells suggesting an additional role of sox21a in promoting ISC proliferation. Is it also something observed here for cad knockdown? Is it true also when cad RNAi is expressed specifically in the EB?

*Response:*

*Thanks for these questions. Zhai et al., 2017[4] had reported that “each esg>GFP+ nest contained a slightly increased number of cells (Fig 2F and 2H), consistent with the notion that Sox21a also promotes a low level of ISC proliferation” (see Fig. 2F and 2H from Zhai et al., 2017[4] listed as below). We checked the midguts of cad knockdown flies. We indeed found that esg^ts^> cad RNAi guts form nests containing an increase in the number of cells similar as seen in esg^ts^> sox21a guts (see Fig 2B-2C and 2E in the revised manuscript). Moreover, the midguts with cad specifically depleted in the EBs by NRE-Gal4 (NRE^ts^) also formed nests containing an increase in the number of cells (see Fig 2F in the revised manuscript).*

*(Fig. 2E-H from Zhai et al., 2017[4])*

7. Regarding the overexpression of cad, why the authors only report its effect after PQ treatment. What is happening in homeostatic conditions? Proposed downstream effectors Sox21a, GATAe, JAK/Stat, all have been tested in normal condition of homeostasis in previous papers Zhai et al., 2015, 2017, Chen et al 2016….

*Response:*

*Thanks for this question. We had checked the midgut phenotypes of cad overexpression flies in homeostatic conditions. Under homeostatic conditions, the ratio of pdm1+esg+ cells or NRE+pdm1+ cells per gut in cad-overexpressed flies and the control flies were quite similar (see S3A-S3F Fig). We think that is reasonable because* *under homeostatic conditions most ISCs in the midguts are quiescent and do not differentiate to produce progenitor cells. Thus, we could not see an obvious* *effect of cad overexpression to prevent the differentiation of these quiescent ISCs. After injury (PQ treatment) these quiescent ISCs become active and begin to produce ECs. Then, we saw the effect that cad overexpression prevents these differentiating cells to produce new ECs.*

In Fig.4G sox21a appears to be upregulated after damage, this is a bit contradictory with the data presented in Fig 1 and Sup1 indicating an upregulation of cad in aged gut and after Pe infection and with data from Chen et al 2016 where a downregulation of Sox21a is observed after DSS damage induction in the gut. The authors should test the impact of PQ and DSS treatments on cad expression level.

*Response:*

*Thanks for this question and suggestion. In our original Fig. 4G we indeed showed a upregulate of sox21a expression in esg+ cells after PQ-induced damage. This is consistent with what was reported by Zhai et al 2017[4]. The Figure 2B from Zhai et al 2017[4] showed that “Oral infection with Ecc15 induced a marked increase of Sox21a-GFP expression in the progenitors (Fig 2B and 2B')” (see Figure 2B from Zhai et al 2017[4] below). Both their and our data showed that Sox21a was induced in intestinal progenitors during regeneration.*

*In the revised manuscript, we have checked the expression levels of cad (indicated by the endogenous CAD-GFP) after PQ and DSS (S1J-K Fig.). Besides, we also checked the expression levels of Sox21a-HA by western blot after PQ induced (S4F Fig). We found that the cad expression level was upregulated during regeneration after either PQ or DSS treatment. We explain that there is a negative feedback relationship between cad and sox21a expression during regeneration. The upregulated cad during regeneration is used to reduce the injury-induced sox21a level to go back to the normal level when the damage gets repaired. In other words, the upregulated CAD prevents sox21a over-activation during regeneration.*

*(Figure 2B from Zhai et al 2017[4])*

8. Since the authors proposed that sox21a is the main target of CAD in the progenitor cells of the midgut it would be interesting to compare their bulk RNAseq data with published RNAseq data for Sox21a overexpression by Zhai et al., 2017. Is there also an upregulation of expected downstream effectors such as pdm1, GATAe, Connectin, armadillo, E-cadherin…

*Response:*

*We appreciate this suggestion. We have checked our RNAseq data of cad RNAi. Unfortunately, we did not find the upregulation of these expected downstream effectors mentioned by this reviewer. We also checked the expression of these genes (including pdm1, GATAe, Connectin, armadillo, and E-cadherin) mentioned by this reviewer mentioned in the RNA-seq data of esg^ts^>UAS-sox21a from Zhai et al., 2017[4]. In fact, we only found Connectin was significantly up-regulated (the expressions of pdm1, GATAe, armadillo, and E-cadherin did not show significant change) from the RNAseq data for Sox21a overexpression by Zhai et al., 2017.*

*Since Zhai et al., 2017[4] did the RT-qPCR using the sorted EB of sox21a mutant midguts and found some downstream effector genes, in the revised manuscript we also checked the mRNA expression of these expected downstream effectors that listed by this reviewer by RT-qPCR using sorted esg+ cells (see Fig 4B). These data showed that GATAe, Connectin, Armadillo, pdm1, and E-cadherin indeed were up-regulated in esg^ts^>cad RNAi midguts compared with control (Fig 4B). We think this may be because that our RNA-seq of cad RNAi were using the whole midguts. The mRNAs from these non-progenitor cells might cover up the real changes of these expected downstream effectors in our RNA-seq.*

9. Since cad appears to be required in the EB to regulate EB-EC differentiation, I am not sure I understand why further genetic interaction experiments and rescue experiments have been done using NREts driver instead of esgts. This specifically true for Sox21a-HA expression upon cad RNAi or UAS-sox21a rescue of UAS-cad-HA experiments in Fig4.

*Response:*

*We appreciate this question. We had used both esg^ts^ and NRE^ts^ to do these genetic interaction experiments and rescue experiments. They showed similar effects. Zhai et al 2015[3] reported that overexpressing Sox21a in EBs but not ISCs induce cell differentiation and “besides inducing progenitor cells to differentiate, Sox21a may also have a role in stem cell division”. Thus, besides using esg^ts^ we also used NRE^ts^ to perform these genetic interaction experiments and rescue experiments.*

10. In rescue and genetic interaction experiments, some controls are missing making impossible to properly interpret the data. For example, in the case of the interaction between cad and Notch pathway, in Fig5 I-L esgts>N RNAi alone is missing. This is important because the double knockdown of N and cad appears to neither give a cad loss phenotype (increase of pdm1+ cells) nor the well described Notch loss phenotype (massive accumulation of ISC/EE and no EC). The conclusion appears more complicated than the one proposed by the authors. Does Notch act downstream, upstream or in parallel of cad?

Similarly, in Fig4 H-K, esgts>UAS-sox21a phenotype alone is missing, in Fig5 A-D esgts>GATAe RNAi condition is missing, in Fig5 E-H NREts>GATAe RNAi condition is missing, in Fig6 D-G esgts>UAS-HopTUM alone is missing, in Fig6 H-K NREts>UAS-HopTUM alone is missing, in Fig6 L-O esgts>stat92E RNAi alone is missing.

*Response:*

*We appreciate these commons and critiques. In the revised manuscript, we have included these controls by both added the representative images and quantification data (see Fig 4I-4M, Fig 5A-5O, S5A-S5E Fig, Fig 6D-6H, and Fig 6I-6M, S6A-S6E Fig). We apologize for not providing the most representative image of midguts with the double knockdown of N and cad in the original Figure 5K. In fact, the phenotype of double knockdown of N and cad is more like the phenotype of N RNAi (by carefully observe our original Figure 5K, several clusters of esg- smalls (EEs) cells indicated by DAPI staining can be found in this image, which is the classic phenotype of N RNAi). We have included an image of N RNAi midguts and a more representative image of midguts with the double knockdown of N and cad to replace the original Figure 5K (see Fig. 5M-5N in the revised manuscript).*

11. The aging experiments are again difficult to interpret at this stage. The authors hypothesis that cad upregulation in aged gut contributes to a decrease of differentiation efficiency. Based on the data, it appears that cad knockdown prevent accumulation of extra esg+ cells as well as Dl+ cells in old guts. The authors do not address general cell death and proliferation in the tissue upon cad knockdown upon aging. Some quantifications should be added to determine whether there is a major effect on ISC survival and division in these conditions. It would be also interesting to evaluate the ratio of esg+ cells to total cells in the different conditions or maybe to induce late MARCM clones to test that indeed there is a change in EC production upon aging and after cad knockdown. Since cad appears to be required only in EB to regulate EC differentiation in young guts, the experiment should be repeated on old guts using NREts as a driver to confirm the cell type specific function of cad upon aging.

Since the upregulation of cad appears limited to R4-R5 regions, is this absence of hyperplasia limited to these regions or is it true all along the midgut? A regional quantification would be helpful. Finally, testing the impact of cad overexpression on aged guts seems to be an obvious experiment.

*Response:*

*We appreciate these commons and have performed additional analyses.*

1) *We checked the general cell death in the midguts of aged flies with cad knockdown by TUNEL staining and did not find a significant increase of cell death in these midguts compared to the aged control (S7E-S7F Fig). Thus, knockdown of cad in esg+ cells led to the reduction of gut hyperplasia phenotype not through inducing ISC death in aged flies.*
2) *We quantified the number of pH3+ cells in midguts of aged flies with cad depletion. We found that knockdown cad in aged midguts led to a reduction in ISC proliferation compared to the aged control (esg^ts^>UAS-GFP) (Fig 7G).*
3) *We have tried but failed to induce normal late MARCM clones in aged animals (most cells will become GFP positive along the whole gut in the aged flies, which may be caused by FLP leak).*
4) *We have used NRE^ts^ to repeat these experiments that we had done using esg^ts^ on old guts (see Fig. 7H). NRE^ts^ showed a similar effect as esg^ts^.*
5) *We have checked the* *hyperplasia condition of the anterior midguts of aged flies with cad knockdown. It seems that knockdown cad can only reduce the hyperplasia in the posterior midguts but not the anterior midguts (see S7A-S7B Fig).*
6) *As expected, cad overexpression* *on aged guts caused more severe gut hyperplasia as indicated by the increase in pH3^+^ cells and esg-GFP^+^ cells (see S7C-S7D Fig).*

12. To extend the proposed model of caudal regulating EB differentiation in EC upstream of the framework composed of JAK-STAT/Sox21a/GATAe to old guts, it would be important to test if some changes of sox21a or GATAe expression are observed upon aging and if cad KD impacts them in old guts.

*Response:*

*We thank this reviewer for this suggestion and have performed additional analyses. We checked the expression changes of SOX21A (endogenous SOX21A-HA) in young and old midguts by western blot analysis (see S4A, S4C Fig). The expression of SOX21A indeed downregulated in midguts of aged flies (S4D-4E Fig). Knockdown cad expression by esg^ts^>cad RNAi significantly reversed the downregulation of SOX21A in aged flies (see S4B, S4C Fig).*

Minor points:

1. In Fig1 C, D , F, G, I, J, L, M it would be helpful to show split channel with GFP only not restricted to insets but for larger views of the region. Representative image of old and young whole midguts or whole post midguts would also help to properly follow CAD-GFP expression

.
*Response:*

*Thanks for this suggestion. We have split the GFP channel for larger views (see Fig. 1A-1H) and included the images of old and young whole midguts (see Fig. S1G-S1I) in the revised manuscript.*

2. There are multiple errors in Fig. 1 legend:
. “(E) Quantification of fluorescence intensity of CAD-GFP in esg-LacZ+ cells of 7-day and 30-day-old Drosophila as shown in (B-C)” instead of (C-D).
. “(H) Quantification of fluorescence intensity of CAD-GFP in Dl+ ISCs of 7-day and 30- day-old Drosophila as shown in (E-F).” instead of (F-G).
. “(K) Quantification of fluorescence intensity of CAD-GFP in Pdm1+ ECs of 7-day and 30-day-old Drosophila as shown in (H-I)” instead of (I-J).
“(N) Quantification of fluorescence intensity of CAD-GFP in Pros+ EEs of 7-day and 30-day-old Drosophila as shown in (K-L)” instead of (M-M).

*Response:*

*Thanks! We have corrected this mistake.*

3. Details on RT-qPCR are lacking in material and methods:
Age of the guts, genotypes, cell sorting protocol and RT-qPCR.

*Response:*

*We apologize for these omissions and have included these details in the Methods section in the revised manuscript.*

4. Ref 22 correspond to mice crypt culture and not aged Drosophila midgut as mentioned in the text.

*Response:*

*We apologize for this error and have corrected it in the revised manuscript.*

1.	Choi YJ, Hwang MS, Park JS, Bae SK, Kim YS, Yoo MA. Age-related upregulation of Drosophila caudal gene via NF-kappaB in the adult posterior midgut. Biochim Biophys Acta. 2008;1780(10):1093-100. Epub 2008/07/29. doi: 10.1016/j.bbagen.2008.06.008. PubMed PMID: 18656526.

2.	Chen J, Xu N, Huang H, Cai T, Xi R. A feedback amplification loop between stem cells and their progeny promotes tissue regeneration and tumorigenesis. eLife. 2016;5. Epub 2016/05/18. doi: 10.7554/eLife.14330. PubMed PMID: 27187149; PubMed Central PMCID: PMCPMC4905741.

3.	Zhai Z, Kondo S, Ha N, Boquete JP, Brunner M, Ueda R, et al. Accumulation of differentiating intestinal stem cell progenies drives tumorigenesis. Nat Commun. 2015;6:10219. Epub 2015/12/23. doi: 10.1038/ncomms10219. PubMed PMID: 26690827; PubMed Central PMCID: PMCPMC4703904.

4.	Zhai Z, Boquete JP, Lemaitre B. A genetic framework controlling the differentiation of intestinal stem cells during regeneration in Drosophila. PLoS Genet. 2017;13(6):e1006854. Epub 2017/07/01. doi: 10.1371/journal.pgen.1006854. PubMed PMID: 28662029; PubMed Central PMCID: PMCPMC5510897.

5.	Du G, Xiong L, Li X, Zhuo Z, Zhuang X, Yu Z, et al. Peroxisome Elevation Induces Stem Cell Differentiation and Intestinal Epithelial Repair. Dev Cell. 2020;53(2):169-84 e11. doi: 10.1016/j.devcel.2020.03.002. PubMed PMID: 32243783.

6.	Dutta D, Dobson AJ, Houtz PL, Glasser C, Revah J, Korzelius J, et al. Regional Cell-Specific Transcriptome Mapping Reveals Regulatory Complexity in the Adult Drosophila Midgut. Cell Rep. 2015;12(2):346-58. Epub 2015/07/07. doi: 10.1016/j.celrep.2015.06.009. PubMed PMID: 26146076.
