# Supplementary figures and images for: Correction: Aging-related upregulation of the homeobox gene caudal represses intestinal stem cell differentiation in Drosophila
Source: PLoS Genet. 2025 Sep 4;21(9):e1011846. doi: 10.1371/journal.pgen.1011846 (PMC12410717; doi:10.1371/journal.pgen.1011846)

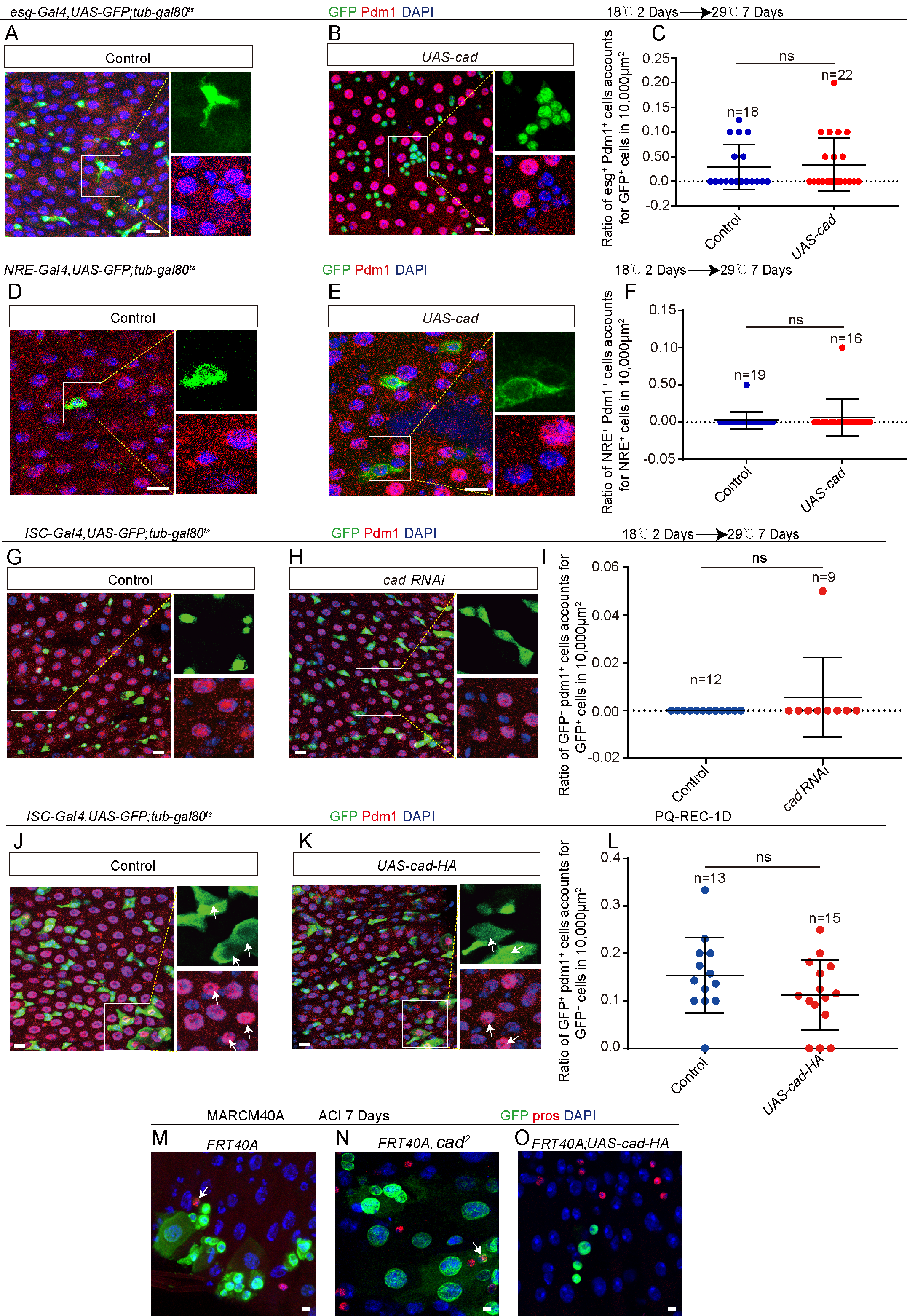

Supplement: S3 Fig — (A-B) Immunofluorescence images of esg-GFP (green) and Pdm1(red) staining with the midgut section from the R4 region of control Drosophila (A, esgts-Gal4 > UAS-GFP) and Drosophila carrying esgts-Gal4 > UAS-cad (B), under normal conditions. esg-GFP (green) represents ISCs and their differentiating cells. Pdm1 staining (red) was used to visualize matured ECs. (C) Quantification of the ratio of esg-GFP+ and Pdm1+ cells per 10,000 μm2 area of the midgut as indicated in (A-B). The number n represents counted ROIs in midguts from each experiment. Each dot corresponds to one ROI (10,000 μm2 area). (D-E) Immunofluorescence images of NRE-GFP (green) and Pdm1(red) staining with the midgut section from the R4 region of control Drosophila (D, NREts-Gal4 > UAS-GFP) and Drosophila carrying NREts-Gal4 > UAS-cad (B), under normal conditions. NRE-GFP (green) represents EBs. Pdm1 staining (red) was used to visualize matured ECs. (F) Quantification of the ratio of NRE-GFP+ and Pdm1+ cells per 10,000 μm2 area of the midgut as indicated in (D-E). The number n represents counted ROIs in midguts from each experiment. Each dot corresponds to one ROI (10,000 μm2 area). (G-H) Immunofluorescence images of ISC-GFP (ISCts-Gal4-driven UAS-GFP; green) and Pdm1 (red) staining with the midgut section from the R4 region of control flies (G, ISCts-Gal4-driven UAS-GFP) and cad-depleted Drosophila by ISCts-Gal4-driven cad RNAi (H). ISC-GFP (green) indicates ISCs. Pdm1 staining (red) was used to visualize differentiating ECs. ISC-GFP+ and Pdm1- cells are ISCs. ISC-GFP- and Pdm1+ cells are mature ECs. (I) Quantification of the ratio of ISC-GFP+ and Pdm1+ cells per 10,000 μm2 area of the R4 region of midguts as shown in (G-H). The number n represents counted regions of interest in midguts from each experiment. Each dot corresponds to one region of interest (ROI = 10,000 μm2 area). (J-K) Immunofluorescence images of ISC-GFP (green) and Pdm1 (red) staining with the midgut treated with PQ-REC-1D. The midgut [file pgen.1011846.s001.tif]
